# Predictive factors for oropharyngeal dysphagia after prolonged orotracheal intubation^☆^

**DOI:** 10.1016/j.bjorl.2017.08.010

**Published:** 2017-09-13

**Authors:** Ana Carolina Martins de Oliveira, Amélia Augusta de Lima Friche, Marina Silva Salomão, Graziela Chamarelli Bougo, Laélia Cristina Caseiro Vicente

**Affiliations:** a Universidade Federal de Minas Gerais (UFMG), Hospital das Clínicas, Saúde do Idoso, Belo Horizonte, MG, Brazil; b Universidade Federal de Minas Gerais (UFMG), Faculdade de Medicina, Departamento de Fonoaudiologia, Belo Horizonte, MG, Brazil; c Hospital Risoleta Tolentino Neves (HRTN), Saúde do Idoso, Belo Horizonte, MG, Brazil; d Hospital Risoleta Tolentino Neves (HRTN), Belo Horizonte, MG, Brazil

**Keywords:** Swallowing disorders, Intratracheal intubation, Intensive care units, Dysphonia, Transtornos de deglutição, Intubação intratraqueal, Unidades de terapia intensiva, Disfonia

## Abstract

**Introduction:**

Lesions in the oral cavity, pharynx and larynx due to endotracheal intubation can cause reduction in the local motility and sensitivity, impairing the swallowing process, resulting in oropharyngeal dysphagia.

**Objective:**

To verify the predictive factors for the development of oropharyngeal dysphagia and the risk of aspiration in patients with prolonged orotracheal intubation admitted to an intensive care unit.

**Methods:**

This is an observational, analytical, cross-sectional and retrospective data collection study of 181 electronic medical records of patients submitted to prolonged orotracheal intubation. Data on age; gender; underlying disease; associated comorbidities; time and reason for orotracheal intubation; Glasgow scale on the day of the Speech Therapist assessment; comprehension; vocal quality; presence and severity of dysphagia; risk of bronchoaspiration; and the suggested oral route were collected. The data were analyzed through logistic regression. The level of significance was set at 5%, with a 95% Confidence Interval.

**Results:**

The prevalence of dysphagia in this study was 35.9% and the risk of aspiration was 24.9%. As the age increased, the altered vocal quality and the degree of voice impairment increased the risk of the presence of dysphagia by 5-; 45.4- and 6.7-fold, respectively, and of aspiration by 6-; 36.4- and 4.8-fold. The increase in the time of orotracheal intubation increased the risk of aspiration by 5.5-fold.

**Conclusion:**

Patients submitted to prolonged intubation who have risk factors associated with dysphagia and aspiration should be submitted to an early speech-language/audiology assessment and receive appropriate and timely treatment. The recognition of these predictive factors by the entire multidisciplinary team can minimize the possibility of clinical complications inherent to the risk of dysphagia and aspiration in extubated patients.

## Resumo

**Introdução:**

Lesões na cavidade oral, faringe e laringe, em virtude de intubação endotraqueal, podem causar redução da motricidade e da sensibilidade local e comprometer o processo da deglutição, determinando disfagia orofaríngea.

**Objetivo:**

Verificar os fatores preditivos do desenvolvimento de disfagia orofaríngea e risco de aspiração em pacientes pós-intubação orotraqueal prolongada internados em uma unidade de terapia intensiva.

**Método:**

Estudo observacional, analítico, de delineamento transversal e retrospectivo de coleta de dados de 181 prontuários eletrônicos, de pacientes submetidos à intubação orotraqueal prolongada. Foram coletadas informações referentes a idade; sexo; doença de base, comorbidades associadas; tempo e motivo da intubação orotraqueal; Escala Glasgow no dia da avaliação fonoaudiológica; compreensão; qualidade vocal; presença de disfagia e a gravidade; risco de broncoaspiração; e via oral sugerida. Os dados foram analisados por meio da regressão logística. Adotou-se o nível de significância de 5% e intervalo de confiança de 95%.

**Resultados:**

A prevalência de disfagia neste estudo foi de 35,9% e de risco de aspiração de 24,9%. O aumento da idade, a qualidade vocal alterada e o grau de comprometimento da voz elevam os riscos de presença em disfagia em 5; 45,4 e 6,7 vezes, respectivamente, e de aspiração em 6; 36,4 e 4,8 vezes. Já o aumento do tempo de intubação orotraqueal elevou em 5,5 vezes o risco de aspiração.

**Conclusão:**

Pacientes submetidos a intubação prolongada que apresentam os fatores de risco relacionados às disfagia e aspiração devem ser submetidos a avaliação fonoaudiológica precoce e receber conduta adequada em tempo hábil. O reconhecimento desses fatores preditivos por toda a equipe multidisciplinar pode minimizar as possibilidades de complicações clínicas inerentes ao risco de disfagia e aspiração em pacientes extubados.

## Introduction

Orotracheal intubation (OTI) is a procedure that allows ventilatory assistance in anesthetized or mechanically-ventilated patients; it may be of short or long-term duration.^1^ Long-term intubation is considered that lasting longer than 48 h.^2^, ^3^ The presence of oro- or nasotracheal tubes in direct contact with airway structures can cause mucosal lesions, mainly due to traumatic and prolonged intubations, the use of large-caliber tubes and the high-pressure tube cuffs.^1^

Lesions in the oral cavity, pharynx and larynx caused by endotracheal intubation, cause reduction in local motility and sensitivity and impair the swallowing process, resulting in oropharyngeal dysphagia.^4^, ^5^, ^6^

The presence of dysphagia can delay the return to oral feeding, increase the risk of pulmonary diseases, and delay hospital discharge.^7^ Additionally, it can trigger problems such as malnutrition and aspiration pneumonia, significantly worsening the hospitalized patient's clinical status.^8^ The incidence of this orotracheal post-intubation complication varies widely from 3% to 83%,^4^, ^5^, ^6^ depending on the method of assessment and characteristics of the studied population.

Early identification of dysphagia is necessary to provide safety to the patient during oral ingestion and, thus, minimize risks of future complications associated with bronchoaspiration.

To that purpose, the bedside clinical Speech Therapist assessment is the most widely used form of swallowing evaluation and, in many hospitals, the only means to investigate the clinical suspicion of a swallowing disorder. It is a non-invasive, fast, low-cost assessment that requires few resources.^9^, ^10^

The Speech Therapist evaluation at the Intensive Care Unit (ICU) aims to identify the possible functional alterations that impair the oral and pharyngeal phases of swallowing.^11^ In addition to the diagnosis of bronchoaspiration, it allows the evaluation of the possibility of oral diet reintroduction, as well as the return of the pleasant sensations associated with eating, concomitant with the use of a feeding tube or not.^12^

Given the importance of oropharyngeal dysphagia after prolonged orotracheal intubation and its implications, studies are needed to identify the factors that contribute to the increased incidence of oropharyngeal dysphagia so that measures can be developed to reduce this incidence and promote strategies for the identification of risk factors by the entire multidisciplinary team, aiming to facilitate early intervention and minimize the occurrence of complications.

Thus, this study aims to verify the predictive factors for the development of oropharyngeal dysphagia and aspiration risk in patients submitted to prolonged orotracheal intubation admitted to an intensive care unit who were submitted to Speech Therapist evaluation at the bedside.

## Methods

This is an observational, analytical, cross-sectional, retrospective study, approved by the Research Ethics Committee of the institution under Opinion n. 51373615.6.0000.5149.

Data were collected from electronic medical records of patients admitted to the Intensive Care Unit (ICU) of a public hospital between November 2012 and June 2015.

The inclusion criteria used in this study were: age older than 18 years; history of prolonged orotracheal intubation (>48 h); having undergone clinical evaluation of swallowing ability at the bedside within the first 48 h after extubation; no history of tracheostomy, neurological or neurodegenerative diseases; no history of oropharyngeal dysphagia prior to hospitalization; esophageal dysphagia and morphological alterations of the head and neck. The medical records of patients for whom Speech Therapist evaluation data and/or information pertinent to the study were incomplete were excluded from the sample.

For the descriptive analysis of the patients’ profile, information was collected on age, gender, underlying disease, associated comorbidities; time and reason for orotracheal intubation; Glasgow scale on the day of the Speech Therapist assessment, comprehension; vocal quality evaluated up to 48 h after extubation through the perceptual-auditory analysis of the sustained vowel /a/ and spontaneous conversation; the presence and severity of dysphagia; risk of bronchoaspiration; and the suggested oral route.

The severity of dysphagia and the risk of aspiration were determined using the Mann Assessment of Swallowing Ability (MASA) protocol. This tool was developed and validated in the English language, translated into Brazilian Portuguese, and is being validated for this language. It includes the motor and sensory evaluation of the structures involved in the swallowing process. Moreover, it evaluates some functions related to the cranial nerves and functional swallowing. It was developed and validated with modified barium swallowing, correctly predicting aspiration in 26 of 28 patients, resulting in a sensitivity of 93%, and is considered a reference standard for bedside evaluation.^13^

Based on the final score of the MASA protocol, dysphagia severity and aspiration risk were determined. Scores between 178 and 200 points indicate absence of dysphagia; scores between 168 and 177 points – mild dysphagia; between 139 and 167 points – moderate dysphagia and values below 138 points – severe dysphagia. For the risk of aspiration, scores between 170 and 200 points indicate absence of risk; between 149 and 169 points – slight risk; between 141 and 148 points – moderate risk and values below 140 points – severe risk.^13^

The functional level of swallowing was determined using the Functional Oral Intake Scale (FOIS),^14^ a widely used tool, both internationally and nationally, with a cross-cultural translation into Brazilian Portuguese,^15^ for the efficient and safe indication of the possible feeding route. It considers the characteristics of the individual's diet by classifying it into seven levels according to the feeding route, dietary limitations and restrictions. These are: Level I: nothing by oral route; Level II: alternative route dependence, minimum oral route of some food or liquid; Level III: alternative route dependence, with a consistent oral supply; Level IV: total oral feeding, but limited to a single consistency; Level V: total oral feeding, with more than one consistency, but requiring special preparations or compensation; Level VI: total oral feeding, with more than one consistency, not requiring special preparations, but with food restrictions; Level VII: total oral feeding, without restrictions.^15^ For statistical purposes, the functional level of the patients’ feeding was grouped: alternative feeding route dependence (FOIS I, II, III), oral feeding limited to a single consistency (FOIS IV), oral feeding with multiple consistencies, but with restrictions (FOIS V and VI) and total oral feeding, without restrictions (FOIS VII).

The functional feeding level represented by the FOIS scale grades was determined after evaluation of the morphofunctional conditions of the stomatognathic system structures and the functional swallowing evaluation. Thus, any alteration caused by the underlying disease or orotracheal intubation was considered when classifying the functional feeding level.

For the statistical analysis, central tendency and dispersion measures were used for the continuous variables and relative frequency distribution for the categorical variables.

The existence of an association between dysphagia and aspiration risk with the univariate and multivariate variables was evaluated using logistic regression analysis, with the associations shown as Odds Ratio (OR). The value of *p* < 0.10 was used as a criterion to include the multivariate variables into the logistic regression model. The explanatory variables analyzed were the patients’ demographic and clinical characteristics.

The variable age was included continuously in the binary logistic regression models as a predictor of dysphagia and aspiration risk, since Pearson's linear correlation coefficients of 0.998 and 0.993 were found, respectively.

To adjust the model having Orotracheal Intubation (OTI) time as the response variable, the Cluster Analysis technique was used, as well as the *K*-means method to divide the number of defined groups. This was initially divided into three groups, the latter characterized by individuals with time of OTI >14 days. However, this was extremely distant from the others and represented only 3.3% of the assessed sample.

Therefore, we chose to divide the sample into only two groups, the first consisting of individuals with OTI time between 2 and 7 days (66.3%) and the second between 8 and 14 days (33.7%).

For all analyses, the significance level was set at 5% and the 95% Confidence Intervals were used. The data collected were tabulated in the Excel program and submitted to univariate and multivariate analyses, with the help of SPSS 23.0 software (Statistical Package for the Social Sciences).

## Results

This study analyzed the medical records of 181 patients who met the inclusion criteria. Table 1 shows the clinical and demographic characteristics of these patients. The sample was predominantly comprised of male individuals (64.1%), with age ranging from 19 to 90 years (47.9 SD 19.3); with chronic diseases being the most prevalent (59.1%) and the main reason for OTI being acute respiratory failure (ARF) (40.3%).

**Table 1:** Demographic and clinical characteristics of patients.

Characteristic	*N* (181)	%
*Gender*		
Female	65	35.9
Male	116	64.1

*Age range*		
18–59 years	125	69.1
60–69 years	27	14.9
70–79 years	18	9.9
Older than 80 years	11	6.1

*Underlying disease*^a^		
Cardiac	14	7.7
Pulmonary	55	30.4
Others	119	65.7

*Comorbidities*^a^		
Absence of comorbidities	59	32.6
Cardiovascular diseases	18	9.9
Chronic diseases	107	59.1
Respiratory diseases	28	15.5
Others	37	20.4

*Reason for OTI*^a^		
Sensory decrease/CRA	38	21.0
ARF	73	40.3
Surgical procedure	57	31.5
Hypovolemic/hemorrhagic shock	20	11.0

*PNM*		
No	141	77.9
Yes	40	22.1

*Glasgow scale*		
9–12	7	3.9
13–15	174	96.1

*Listening comprehension*		
Can follow simple conversation/instruction with repetition	15	8.3
Can follow normal conversation with a little difficulty	10	5.5
No abnormality	156	82.6

*Vocal quality*		
Harsh, wet voice, difficulty in controlling pitch and intensity	77	42.5
Slight impairment, mild hoarseness	35	19.3
No abnormality	69	38.1

a Non-exclusionary variables. *N*, number of participants; OTI, orotracheal intubation; CRA, cardiorespiratory arrest; ARF, acute respiratory failure; PNM, pneumonia.

Among the 181 patients; 96.1% had a Glasgow Coma Scale score >13; 82.6% of the patients were alert and cooperative at the time of the evaluation and 42.5% had hoarse, wet vocal quality and difficulty in controlling pitch and intensity. Regarding OTI time, the minimum was 2 days and the maximum, 21 days (6.3 SD 3.7).

Table 2 shows the results of the clinical evaluation of swallowing. The prevalence of dysphagia in the elderly, aged 60 years or older, was 44.4% and the risk of aspiration was 33.9%. In the non-elderly adults they were, respectively, 32% and 20.8%. After the functional evaluation of swallowing, the consistency that was predominantly suggested by speech therapists was the pureed one (47%).

**Table 2:** Result of the Speech Therapist assessment of the swallowing ability.

	*N* (181)	%
*Dysphagia*		
Present	65	35.9
Absent (178–200)^a^	116	64.1

*Dysphagia severity (N* *=* *65)*		
Mild (168–177)^a^	22	33.8
Moderate (139–167)^a^	37	56.9
Severe (<138)^a^	6	9.2

*Risk of aspiration*		
No risk (170–200)^a^	136	75.1
Risk of aspiration	45	24.9

*Risk of aspiration severity (N* *=* *45)*		
Mild (149–169)^a^	16	35.6
Moderate (139–148)^a^	18	40.0
Severe (<140)^a^	11	24.4

*Post-assessment FOIS*		
I	1	28.2
II	46	0.5
III	14	25.4
IV	41	7.7
V	11	22.7
VI	17	6.1
VII	51	9.4

*Grouped FOIS*		
I, II, III	98	54.1
IV	14	7.7
V, VI	52	28.7
VII	17	9.5

*Post-assessment suggested oral route*		
Suspended	52	28.7
Restricted Liquid	6	3.3
Complete Liquid	5	2.8
Pureed	85	47.0
Soft	15	8.3
Free	18	9.9

*N*, number of participants; FOIS, Functional Oral Intake Scale.

a Scores based on the MASA (Mann Assessment of Swallowing Ability) protocol.

The predictive factors for the risks of dysphagia and aspiration are shown in Table 3, Table 4, and only the final model variables that had a statistically significant *p* value were described. Thus, it is worth noting that the other variables that were not associated with dysphagia and aspiration were not described in the tables. Older age, altered vocal quality and the degree of voice impairment increased the risks of the presence of dysphagia and aspiration. A longer period of orotracheal intubation also increased the risk of aspiration.

**Table 3:** Final multivariate logistic regression model associated with the presence of dysphagia.

Variables	OR (95% CI)	*p*-value^a^
Age	1.05 (1.02–1.08)	0.001
Vocal quality – no abnormality (reference)	1	–
Harsh, wet voice, difficulty in controlling pitch and intensity	45.40 (8.90–231.55)	<0.001
Slight impairment, mild hoarseness	6.72 (1.28–36.81)	0.028

OR, Odds Ratio; CI, Confidence Interval.

a Significant values (*p* < 0.05).

**Table 4:** Final multivariate logistic regression model associated with the presence of aspiration.

Variables	OR (95% CI)	*p*-value^a^
Age	1.06 (1.03–1.10)	<0.001
Time of OTI 8–14 days	5.78 (2.03–16.44)	0.001
Vocal quality – no abnormality (reference)	1	–
Harsh, wet voice, difficulty in controlling pitch and intensity	36.41 (5.72–234.12)	<0.001
Slight impairment, mild hoarseness	4.80 (0.66–34.44)	0.028

OR, Odds Ratio; CI, Confidence Interval; OTI, Intratracheal intubation.

a Significant values (*p* < 0.05).

The rate of correct identification of the risk for presence of dysphagia was 83.8%; the *R*^2^ value obtained was 0.629, indicating that the model explains 62.9% of the variability of dysphagia presence. On the other hand, the rate of correct identification of aspiration risk was 87.8%; the *R*^2^ value obtained was 0.624, indicating that the model explains 62.4% of the variability of the aspiration risk.

It was also noted that the only variable that showed an association with the duration of orotracheal intubation was the risk of aspiration. Thus, individuals who remained intubated for prolonged periods of time, between 8 and 14 days, had a 5.5-fold increased chance of aspiration than those who remained intubated for shorter periods (OR = 5.50, 95% CI: 2.59–11.66; *p* = 0.000).

## Discussion

Dysphagia is defined as the difficulty or inability to safely and efficiently transfer food and fluids from the oral cavity to the stomach, and is usually observed in critically-ill patients who required orotracheal intubation for mechanical ventilation.^7^ As a result of the swallowing disorder, the patient may have risks of dehydration, malnutrition, aspiration of secretions or food, and death.^6^, ^16^

The orotracheal tube can cause laryngeal complications related to duration of intubation, orotracheal tube size and cuff pressure, that may cause irreversible sequelae to the patient.^17^ Among the possible complications resulting from prolonged orotracheal intubation, oropharyngeal dysphagia is notable.

The incidence of oropharyngeal dysphagia obtained in this study was 35.9% and the risk of aspiration was 24.9%. The literature shows a wide variability regarding dysphagia incidence, which can be attributed to the tools used in the diagnosis, either through instrumental or clinical evaluation, as well as related to the characteristics of the study population. Some studies have demonstrated an occurrence of dysphagia greater than 20%, either through clinical and/or instrumental evaluations^2^, ^7^, ^18^ and the incidence of aspiration, obtained through videofluoroscopy, between 20% and 56% of patients being mechanically ventilated for at least 48 h.^2^, ^19^ Considering the risks patients are prone to have when dysphagia is present and the clinical complications inherent to bronchoaspiration, this study found a high prevalence rate of dysphagia and aspiration risk. Therefore, it is of utmost importance that the intensive care team be aware of the risk factors and aim to minimize such prevalence rates.

It is known that the instrumental assessment of swallowing, performed by videofluoroscopy or videoendoscopy, is the most reliable method of swallowing assessment.^10^ However, it is not always performed in clinical practice due to patients’ clinical instability and the inability of patients to cooperate^20^ or the unavailability of these resources at the service. The hospital where the present study was carried out belongs to the public health system, and the instrumental evaluation of swallowing is not an available resource, since videofluoroscopy is not a procedure offered by the Brazilian Unified Health System (Sistema Único de Sáude – SUS). Moreover, as the study sample came from the ICU, moving the patients to undergo this test could be contraindicated, due to their clinical instability. Therefore, the ideal situation would be performing the evaluation in the ICU, with transportable equipment such as the swallowing videoendoscopy. However, the hospital does not have an Otorhinolaryngology Service to perform this procedure, which illustrates the importance of having such resources available, including in high-complexity hospital services.

Post-extubation dysphagia is considered a multifactorial condition, which may occur due to oropharyngeal muscular inactivity, glottic lesion, mucosal inflammation leading to loss of tissue architecture, vocal fold ulcerations, and prolonged narcotic and anxiolytic drug effects that may attenuate airway protective reflexes.^6^, ^21^

This study revealed that age was a predictive factor for both dysphagia and aspiration risk, increasing the risk by five and six-fold, respectively. In addition, there was a higher prevalence of dysphagia and risk of aspiration in the elderly than in non-elderly adults. However, there is no consensus in the literature regarding the predisposition to dysphagia or the risk of aspiration due to age. Some studies have shown an increased risk of post-extubation aspiration in patients 55 years of age or older.^21^, ^22^, ^23^ Other authors affirm that elderly patients do not have an increased risk of swallowing disorders after extubation when compared to the young population.^21^, ^24^ However, the age variable seems to significantly affect the resolution of dysphagia, since the return to oral feeding in the elderly tends to be delayed.^21^, ^23^, ^25^

Regarding intubation time, it was observed that patients who remained intubated for 8–14 days showed a 5.5-fold higher risk of aspiration. This value is three times greater than that found in a recent study, which reported that the risk of developing post-extubation dysphagia was 1.82-fold higher in subjects intubated for 7 days.^26^ Another study suggests that the higher frequency of predictive signs of aspiration risk is present even earlier, after only six days of intubation.^27^ Time of intubation was also considered an independent predictor of dysphagia in other studies.^7^, ^28^, ^29^, ^30^ The presence of the orotracheal tube causes alterations in the chemoreceptors and/or mechanoreceptors located in the pharyngeal and laryngeal mucosa, involved in the swallowing reflex.^31^ Alterations from a mechanical cause are directly related to the duration of intubation and the size of the endotracheal tube, since these may cause mucosal inflammation leading to loss of architecture, oropharyngeal muscular atrophy due to lack of use during intubation, reduction of proprioception and laryngeal sensitivity.^6^

Airway complications secondary to orotracheal intubation are frequent and the symptoms are usually of short-duration, but in some cases, the lesions may be severe and permanent, and involve laryngeal and tracheal structures. Occasionally they requiring a surgical treatment.^17^ According to Mota et al., the most frequent alterations are: edema, ulcers, lacerations, cartilaginous trauma, dysphonia, vocal fold paresis or paralysis, polyps, granulomas and laryngeal stenosis. One of the most frequent symptoms observed by patients submitted to orotracheal intubation is hoarseness. We noted some degree of hoarseness in 61.9% of our patients with, which is above the reported incidence of 14.4–50%.^17^ It is believed that the patients’ clinical conditions may have influenced this, since most of them had chronic comorbidities, such as diabetes mellitus and systemic arterial hypertension. Additionally, it is known that such diseases make patients more vulnerable to mechanical damage and cuff pressure of the orotracheal tube due to peripheral diabetic neuropathy and arterial atherosclerotic alterations of the larynx.^32^ Normally, resolution of dysphonia occurs between 24 and 48 h; however, if symptom persist for more than 72 h, vocal fold lesions should be suspected.^33^

Alterations in glottic function are also related to dysphagia, as the vocal folds are responsible for both phonation and the protection of the lower airways. Glottic coaptation and impairment of laryngeal sensitivity result in the risk of aspiration. In this study, there was a significant association between altered vocal quality and dysphagia and risk of aspiration.

Thus, it is verified that patients submitted to prolonged intubation who manifest glottic dysfunction have a considerably increased chance of having dysphagia and aspiration. The present study showed that patients who had an altered vocal quality had a 45-fold higher chance of developing dysphagia. This odds ratio is greater than twice that found in the literature, of which risk of dysphagia was 20-fold higher.^27^ Therefore, it is emphasized that the intensive care team should be attentive to the symptom of post-extubation dysphonia, since the vocal alteration makes the patients susceptible to the risk of dysphagia and aspiration. Thus, it is important that these patients be evaluated early, preferably by a Speech Therapist and by an otorhinolaryngologist, in order to minimize the impact triggered by orotracheal intubation.

Finally, it is worth mentioning the limitations of this study. First, the difficulty caused by the lack of a standardized swallowing protocol for intubated patients, with the possibility of determining the severity of dysphagia to be used in the hospital, is highlighted. Therefore, it was necessary to use the MASA protocol, which allows classifying the dysphagia severity and the aspiration risk through means of scores. Secondly, the type of evaluation used to determine the risk indicators for dysphagia and aspiration, which was made exclusively through the clinical and Speech Therapist assessment. It is known that the video-fluoroscopy is considered the gold standard for swallowing evaluation, so there is a possibility that the incidence of dysphagia might be higher, due to the probability that patients with silent aspiration were not identified at the bedside assessment.

Despite such limitations, this study has important clinical effects as it allows the intensive care team, mainly the physicians, to evaluate the risk factors that make patients prone to clinical complications, such as dysphagia. Moreover, it helps in the quick identification of these patients, so they can receive the adequate Speech Therapist intervention at an early stage.

Thus, it is suggested that patients submitted to prolonged intubation who have the identified risk factors for dysphagia and aspiration should be submitted to early Speech Therapist assessment. The recognition of these predictive factors by the entire multidisciplinary team can minimize the possibility of clinical complications inherent to the risk of dysphagia and aspiration in extubated patients.

## Conclusion

The predictive factors that increased the chance of dysphagia and aspiration after orotracheal intubation were age, vocal quality alteration, and voice impairment degree. A time of intubation longer than seven days was found to be a predictor only for the risk of aspiration.

## Conflicts of interest

The authors declare no conflicts of interest.
